# New periodic-chaotic attractors in slow-fast Duffing system with periodic parametric excitation

**DOI:** 10.1038/s41598-019-46768-7

**Published:** 2019-08-01

**Authors:** Xianghong Li, Yongjun Shen, Jian-Qiao Sun, Shaopu Yang

**Affiliations:** 1 Department of Mathematics and Physics, Shijiazhuang Tiedao University, Shijiazhuang, 050043 China; 2 Department of Mechanical Engineering, Shijiazhuang Tiedao University, Shijiazhuang, 050043 China; 3 School of Engineering, University of California, Merced, CA 95343 USA

**Keywords:** Nonlinear phenomena, Mechanical engineering

## Abstract

A new type of responses called as periodic-chaotic motion is found by numerical simulations in a Duffing oscillator with a slowly periodically parametric excitation. The periodic-chaotic motion is an attractor, and simultaneously possesses the feature of periodic and chaotic oscillations, which is a new addition to the rich nonlinear motions of the Duffing system including equlibria, periodic responses, quasi-periodic oscillations and chaos. In the current slow-fast Duffing system, we find three new attractors in the form of periodic-chaotic motions. These are called the fixed-point chaotic attractor, the fixed-point strange nonchaotic attractor, and the critical behavior with the maximum Lyapunov exponent fluctuating around zero. The system periodically switches between one attractor with a fixed single-well potential and the other with time-varying two-well potentials in every period of excitation. This behavior is apparently the mechanism to generate the periodic-chaotic motion.

## Introduction

Chaos is a typical motion in nonlinear systems, which is characterized by the unpredictable behavior and extreme sensitivity to initial conditions^1^. Because of the broad-band and noise-like spectrum, chaotic motions are useful in various engineering applications, such as secure communication, image encryption, random bit generation, radar and sonar systems^2–5^. On the other hand, chaos should be avoided in order to separate periodic motion from chaos by applying small perturbations^6^. Among chaotic systems, the Duffing oscillator has played a very important role and was the first chaotic system observed experimentally^7^. The Duffing oscillator with single-well, two-well and three-well potentials had been extensively studied analytically and numerically in engineering, physics, electronics, neurology, biology and other fields^8–18^. The Duffing systems subject to different external excitations were investigated^19,20^, where the necessary conditions for chaos based on both homoclinic and heteroclinic bifurcations were obtained. A Duffing system subject to two external excitations was discussed, and parametric threshold values for chaos were identified in^21^. The Duffing equation with damping and external excitations was also investigated, and the criteria of existence of chaos were found in^22^. The rich dynamical behaviors and bifurcations of the Duffing equation with parametric and external excitation were reported in^23^.

Strange nonchaotic attractors (SNAs) are geometrically complicated, exhibit no sensitive dependence on initial conditions and possess non-positive Lyapunov exponent^24–30^. Grebogi *et al*.^24^ found that quasi-periodically driven dynamical systems admitted SNAs in parameter regions of positive Lebesgue measure. Many other studies on SNAs in quasi-periodically driven systems were later reported^31–35^. On the other hand, the SNA in dynamical system without quasi-periodic excitation is becoming more and more attractive. Although the SNA in an autonomous four-dimensional mapping was reported in^36^, the accurate calculation of the maximum Lyapunov exponent was not confirmed^37^. Recently, the SNA was observed in a periodically driven nonlinear three degree-of-freedom vibro-impact system with symmetric two-sided rigid constraints^38,39^. We should point out to our best knowledge that the dynamic responses involving both chaotic and nonchaotic characteristics are not available in the literature.

In this paper, we consider the Duffing system with periodically slowly time-varying stiffness, which exhibits this chaotic and nonchaotic switching dynamics, and is called the periodic-chaotic motion. The rest of the paper is organized as follows.

## The System and its Complexity

Consider the Duffing system with periodical parametric excitation

1 $$\{\begin{array}{rcl}\dot{x} & = & y\\ \dot{y} & = & (a\,\cos \,\omega t)x-{x}^{3}-by\end{array}$$

where *b* is the damping coefficient, and *a* and ω are the amplitude and frequency of the excitation. The stiffness is periodically time-varying. It is positive in one half period and negative in the other half. Hence, the equilibrium state (0, 0) is stable in one half period and unstable in the other half. Furthermore, the non-zero equilibrium states exist for the first half period when *a*cosω*t* >0 and move on the *x*-axis in the region [$-\,\sqrt{a},\,\sqrt{a}$]. These characteristics are the reason for highly complex and unusual dynamic responses of the system, including the new phenomenon of periodic-chaotic motions.

**Figure 1:**
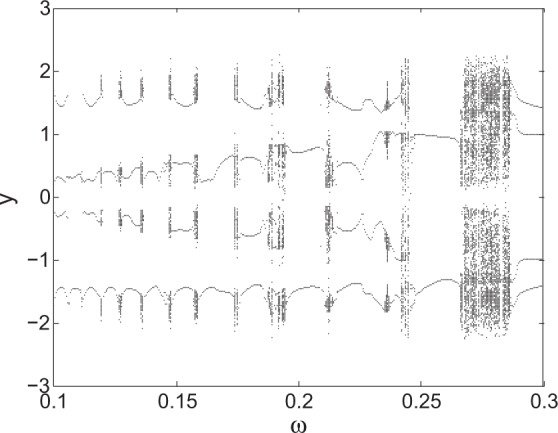
Bifurcation diagram with respect to excitation frequency ω. *a* = 6.25, *b* = 0.3.

**Figure 2:**
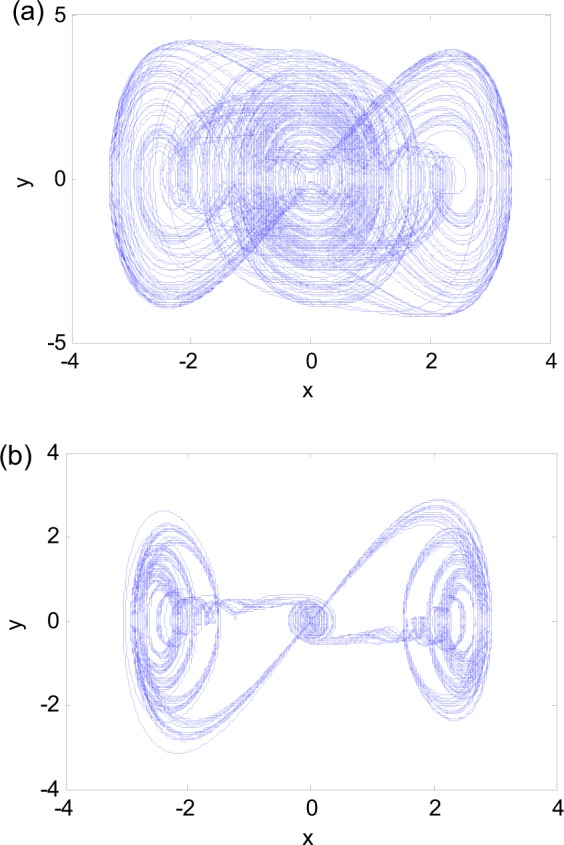
Phase diagrams of chaotic attractors for (**a**) ω = 0.7 and (**b**) ω = 0.1355.

**Figure 3:**
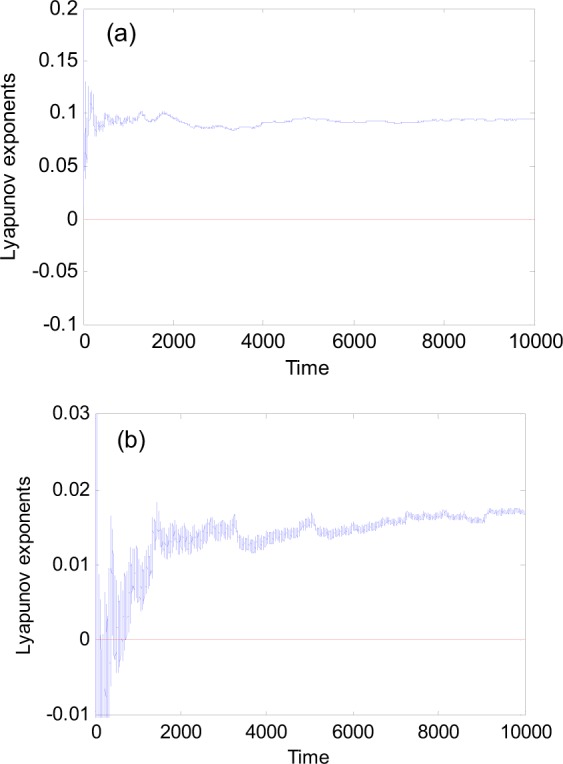
The maximum Lyapunov exponents of chaotic attractors for (**a**) ω = 0.7 and (**b**) ω = 0.1355.

Examples of the system response are shown in Fig. 1, where the bifurcations of *y* with respect to ω with *a* = 6.25 and *b* = 0.3. When ω = 0.7 and ω = 0.1355, chaotic attractors exist as shown in the phase plane plots of Fig. 2(a,b). Their maximum Lyapunov exponents change from 0.1 to less than 0.02 presented in Fig. 3(a,b).

These examples clearly show the rich dynamics of the system and imply the difficulty to study it analytically. For this reason, the paper mainly presents a series of numerical investigations of the system.

## Fixed-point Chaos

**Figure 4:**
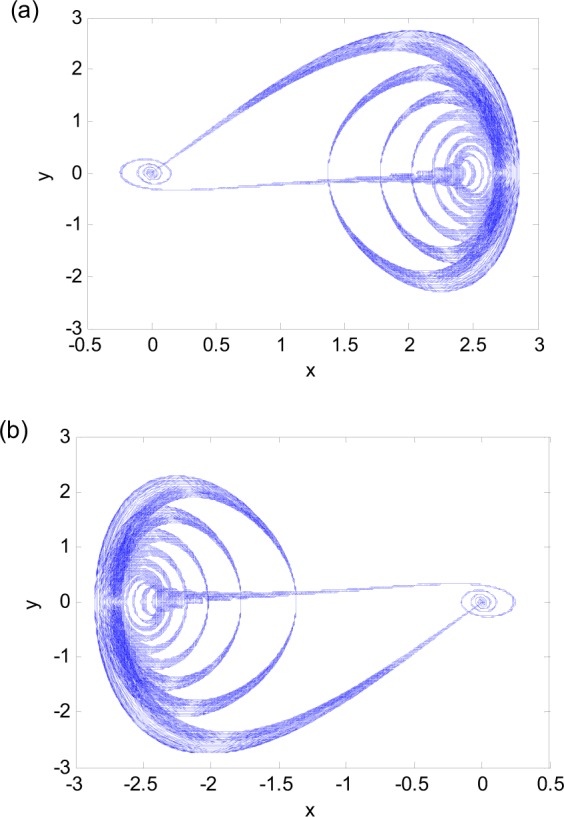
Two coexisting fixed-point chaotic attractors for $a=6.2575$ and $\omega =0.076$_._ (**a**) Initial point (0.1, 0.1). (**b**) Initial point (−0.1, −0.1).

**Figure 5:**
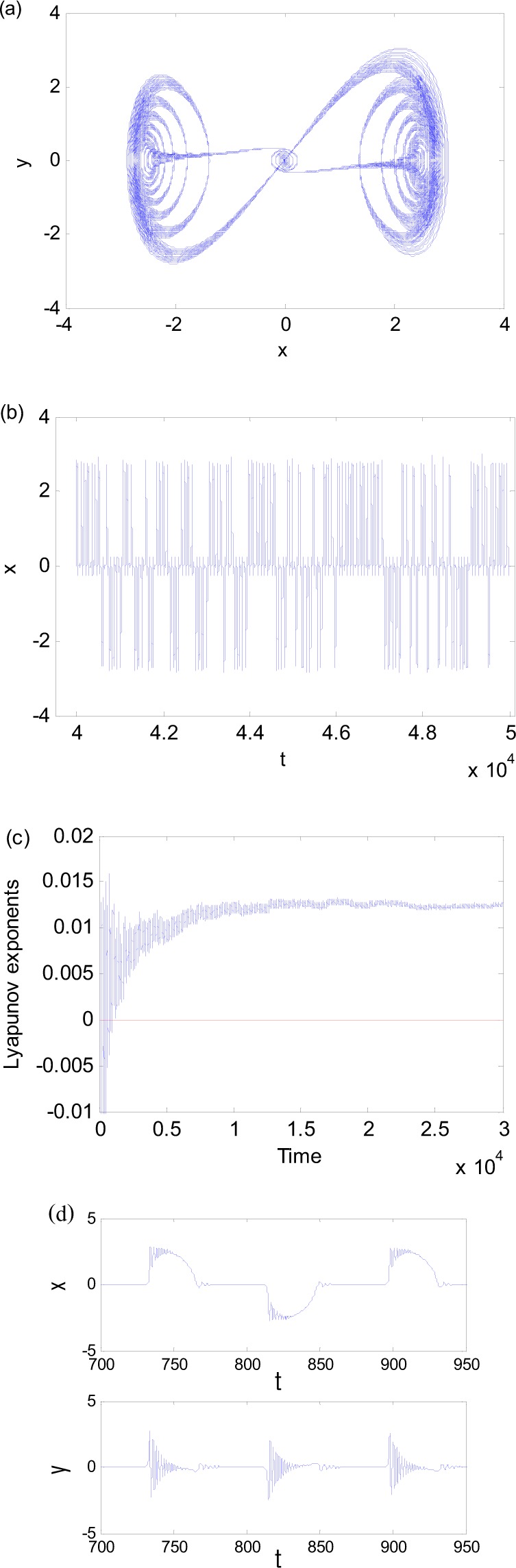
Fixed-point chaos for $a=6.25,\omega =0.076$. (**a**) Phase diagram. (**b**) Time history. (**c**) The maximum Lyapunov exponent. (**d**) Enlargement of time history.

As a special case of periodic-chaotic motions, we consider a new phenomenon of fixed-point chaotic motion. For $\omega \ll 1$, Eq. (1) may become a slow-fast system with two time scales. When the parameters are taken as $\omega =0.076$, $a=6.2575$ and $b=0.3$, two chaotic attractors coexist. The phase diagrams of the chaos starting from two initial points (0.1, 0.1) and (−0.1, −0.1) are shown in Fig. 4(a,b). When *a* changes to 6.25 while other parameters are kept the same, the two coexisting fixed-point chaotic attractors merge. The phase diagram, time history, and maximum Lyapunov exponent of the resulting attractor are presented in Fig. 5(a–c). The maximum Lyapunov exponents of these attractors are positive and indicate that they are indeed chaotic.

These attractors, however, are different from the classic chaos. The “randomness” of these attractors is not obvious. The trajectories in Figs 4 and 5 seem to be anchored at the fixed point (0, 0), which attracts in one half of the period when *a*cosω*t* < 0 and expels in the other half when *a*cosω*t* > 0. The time history of *x* in Fig. 5(d) clearly shows the pattern. This is the reason that we call this phenomenon as the fixed-point chaos.

To further study the mechanism of the fixed-point chaos, we examine the bifurcation behavior of the slow-fast system. For $\omega \ll 1$, the periodic excitation $f=a\,\cos \,\omega t$ changes slowly between $[-\,a,\,a]$. We treat *f* approximately as a contant and use it as a bifurcation parameter of the following autonomous system

2 $$\{\begin{array}{rcl}\dot{x} & = & y\\ \dot{y} & = & fx-{x}^{3}-by\end{array}$$

**Figure 6:**
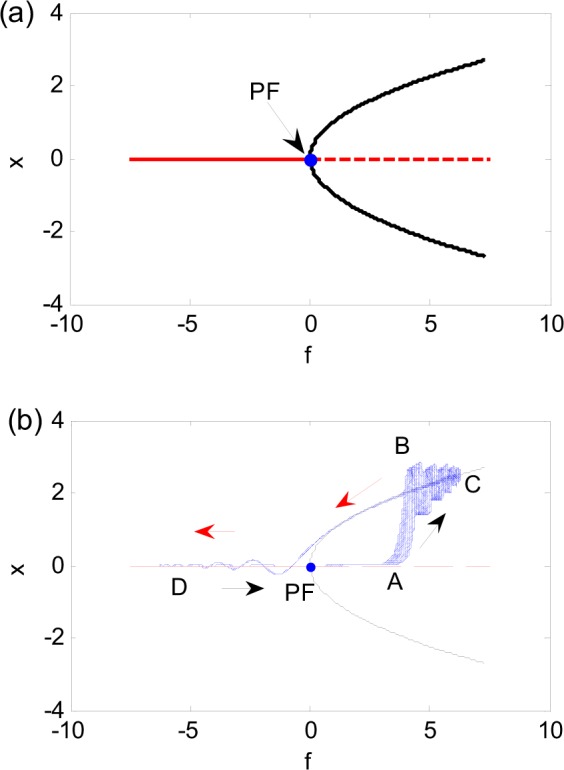
Generation mechanism of fixed-point chaos. (**a**) Bifurcation diagram. (**b**) Overlapping the bifurcation diagram in (**a**) with the transformation diagram of Fig. 4(a).

Figure 6(a) presents the bifurcation diagram of Eq. (2) for *b* = 0.3, where the equilibrium (0, 0), is always stable for $f < 0$, and unstable for $f > 0$, expressed by solid and dash red lines respectively. The solid black line represents the stable equilibria $(\,\pm \,\sqrt{f},\,0)$ for $f > 0$. Therefore, pitchfork bifurcation happens at $f=0$ denoted as PF.

We take Fig. 4(a) as an example to explain the mechanism of the fixed-point chaos. Map the time history of *x*(*t*) in Fig. 4(a) as the function of $f=6.2575\,\cos (0.076t)$, called transformation diagram, and overlap the results with Fig. 6(a). Figure 6(b) shows the changing pattern of the regular motion and chaos. To the left of point A, the trajectory may stay at the quiescence state (QS). To the right of point A, the trajectory is drawn by the upper stable branch and jumps to point B so as to form spiking state (SP). Thus, Point A is a turning point from the quiescence state (QS) to the spiking state (SP).

With the increase of the excitation, the system may have the SP state around the stable branch. Because the equilibrium on the upper branch is stable, the oscillation amplitude of the SP state is gradually damped. When the excitation reaches the maximum amplitude 6.2575, i.e. point C shown in Fig. 6(b), the trajectory may change direction and move on the same stable branch to stay on the QS with the decrease of the excitation. When trajectory arrives at the bifurcation point PF, it may be attracted by the left stable point, and begins to approach the stable equilibrium point (0, 0). The minimum of the excitation with amplitude −6.2575 is denoted as point D. At this point, the trajectory turns around to move back to point A after passing the equilibrium (0, 0). Then, the equilibrium (0, 0) becomes unstable and expels the system toward points A and C. The randomness shown in the collection of trajectories is most likely due to the fact that when the system is attracted to the stable equilibrium (0, 0), the response variables *x* and *y* are non-zero and very small. In digital computations or experiments, such small numbers are practically random. Hence, the trajectories leaving the equilibrium (0, 0) all have different initial conditions for each period. The sensitivity to initial conditions shown in this system is clearly a property of chaos. This phenomenon is also common in slow-fast systems with switches between different attractors of the fast subsystem. Although the motion of the system in every period is regular, the totality of the responses constitutes a chaotic motion.

The fixed-point chaos is a bursting oscillation such that the SP state is coupled with the QS. This is typical with the slow-fast system. The hysteresis loop in the *x*-*f* plane in Fig. 6(b), indicating the memory effect of the system, usually exists in the slow-fast system^40^.

The mechanism for the big fixed-point chaos in Fig. 5 is similar to that in Fig. 4. The difference is that the trajectory randomly visits the left and right branches in Fig. 5(a), as indicated by the time histories in Fig. 5(b). The randomness is again due to the smallness of the system response when it leaves the stable equilibrium (0, 0).

## Fixed-point Strange Nonchaotic Attractor

**Figure 7:**
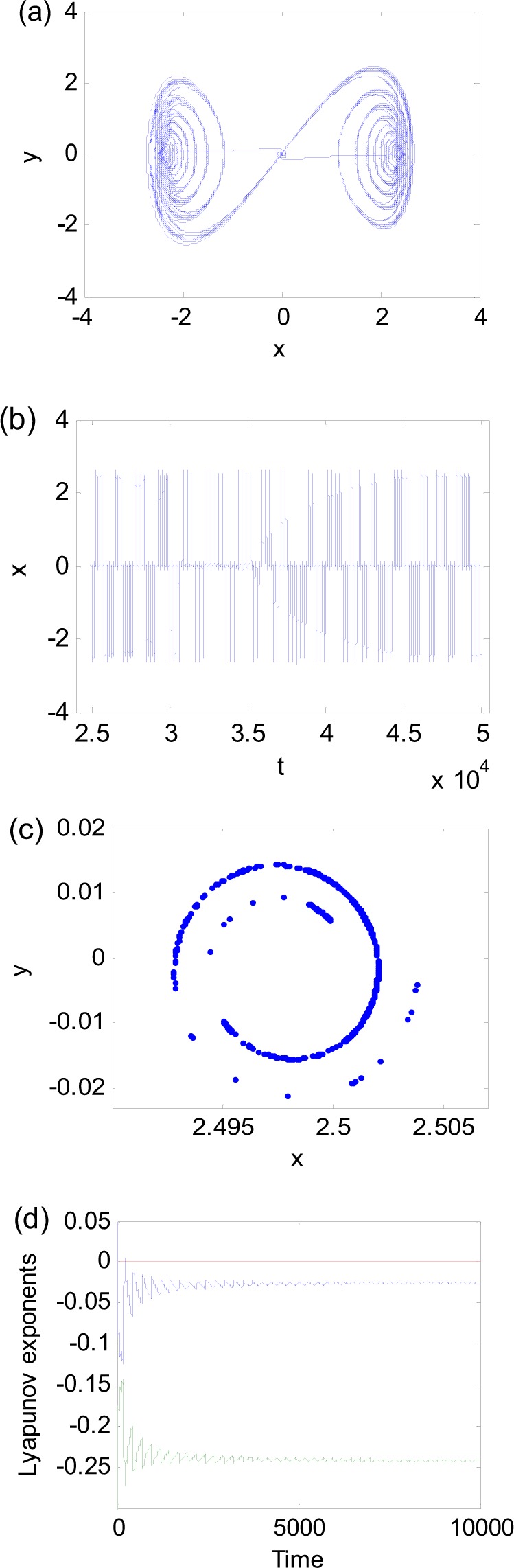
Nonchaotic attractors for $a=6.25,\omega =0.025$ and $b=0.268012$. (**a**) Phase diagram. (**b**) Time history. (**c**) Poincare section. (**d**) Lyapunov exponent.

As the excitation frequency decreases, the fixed-point chaos may turn into another attractor. Figure 7 presents the oscillation of Eq. (1) for parameters $a=6.25$, $\omega =0.025$, and $b=0.268012$. The phase diagram in Fig. 7(a), time history in Fig. 7(b), and Poincare section in Fig. 7(c) are similar to those of fixed-point chaos. However, the maximum Lyapunov exponent in Fig. 7(d) is not positive. Therefore, the response is nonchaotic. We would like to call it a strange nonchaotic attractor because the attractor is not periodic, quasi-periodic and chaotic. Strange nonchaotic attractors are mostly reported in the systems subject to excitations with two incommensurate or irrational frequencies^24–30^. It should be pointed out that there is only one frequency involved in this system. Because this nonchaotic attractor periodically visits the fixed point, we call it the fixed-point strange nonchaotic attractor.

**Figure 8:**
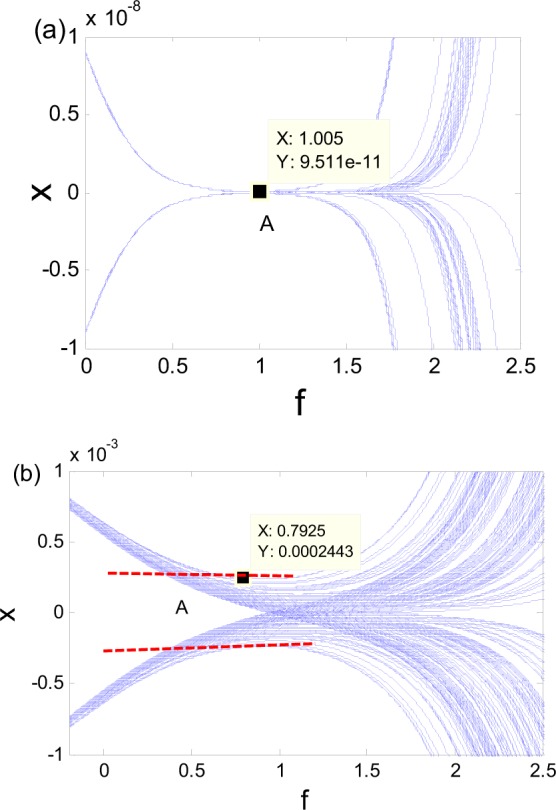
Oscillation near turning point A under *f* = *a*cos*ωt*. (**a**) Fixed-point strange nonchaotic attractor for $a=6.25$, $\omega =0.025$, and $b=0.268012$. (**b**) Fixed-point chaotic attractor for $a=6.25,\omega =0.076$, and $b=0.3$.

To further examine the effect of the excitation frequency, we rewrite Eq. (1) in an extended state space as

3 $$\{\begin{array}{rcl}dx/d\theta  & = & y/\omega \\ dy/d\theta  & = & [(a\,\cos \,\theta )x-{x}^{3}-by]/\omega \\ d\theta /d\theta  & = & 1\end{array}$$

where $\theta =\omega t$. The subsystem consisting of *x* and *y* is fast, and the system associated with $\theta$ is slow. The equilibrium (0, 0) of the fast subsystem is stable for $\cos \,\theta  < 0$ and unstable for $\cos \,\theta  > 0$. The eigenvalues are ${\lambda }_{1,2}=(-\,b\pm \sqrt{{b}^{2}+4a\,\cos \,\theta })/2\omega$, and $\mathrm{Re}({\lambda }_{1,2})$ determines the stability of the equilibrium (0, 0). Parameters *b* and $\omega$ may directly affect the real part of eigenvalues. Because $\omega$ is in denominator, the variation of $\omega$ may lead to a large change of the real part of the eigenvalues. For example, substituting the parameters of Figs 5(a) and 7(a) into $\mathrm{Re}(\lambda )$, we obtain ${{\rm{Re}}(\lambda )|}_{chaos}=-\,1.97$ and ${{\rm{Re}}(\lambda )|}_{nonchaos}=-\,5.36$. It is obvious that the attraction of the stable equilibrium (0, 0) for nonchaos is much larger than that of the chaos. The displacement range of the turning point A in nonchaos is not exceeding 2·10^−9^ as shown in Fig. 8(a), while the range of point A in chaos falls in the region (−0.000243, 0.0002443) as shown in Fig. 8(b). The increase of attraction of stable equilibrium (0, 0) makes the range of the initial points entering SP in nonchaos much less than that in chaos. Although the extreme sensitivity to initial conditions exists in nonchaos presented in Fig. 8(a), the maximum Lyapunov exponent of whole trajectory is negative due to its local and transient property.

**Figure 9:**
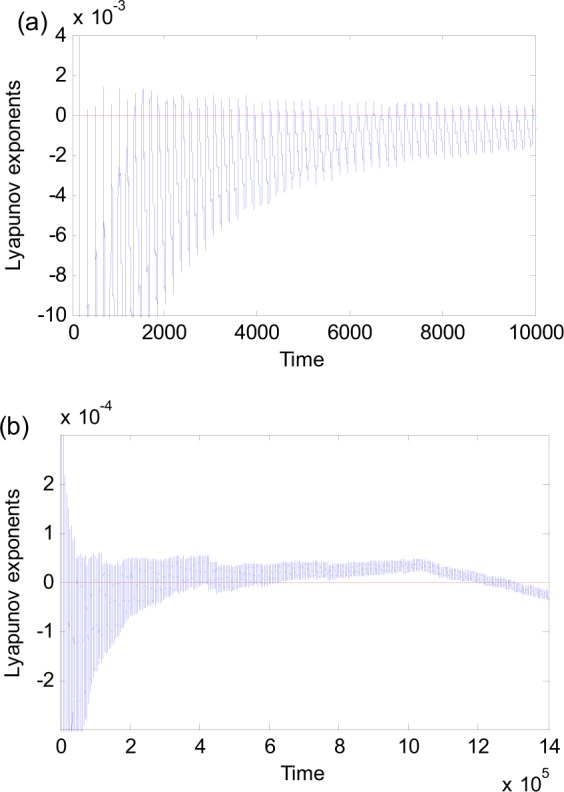
The maximum Lyapunov exponent of critical behavior between chaos and nonchaos for $a=6.249984719222$, $\omega =0.0365445$, and $b=0.268012$. (**a**) Time is 10000. (**b**) Time is 1400000.

Because the equilibrium (0, 0) converges and diverges in every period, the maximum Lyapunov exponent oscillates with the same frequency as that of the frequency. What is the maximum Lyapunov exponent of the critical behavior between chaos and nonchaos? For $a=6.249984719222$, $\omega =0.0365445$, and $b=0.268012$, the maximum Lyapunov exponent is plotted in Fig. 9(a,b) over different time intervals. We find that the maximum Lyapunov exponent of the critical behavior between fixed-point chaos and nonchaos always oscillates around zero. Such a critical solution may exist in a range of the system parameters, not at a point.

## Periodic-Chaotic Oscillations

**Figure 10:**
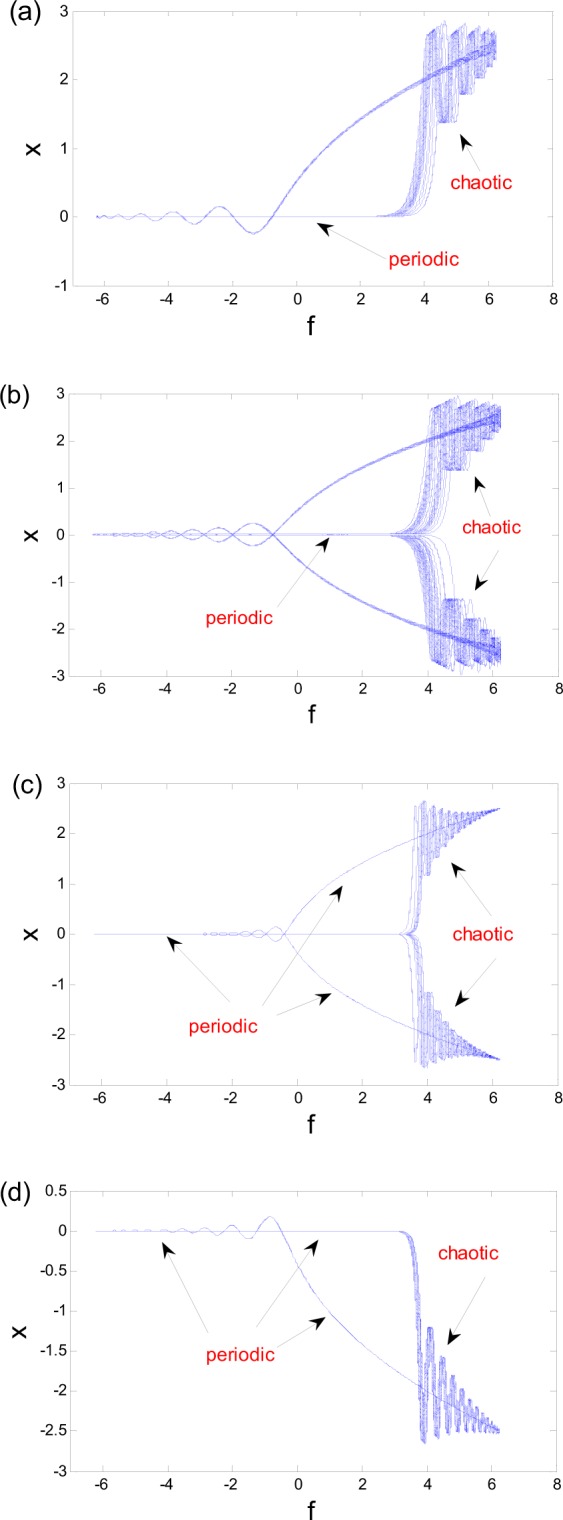
Transformation diagrams, under $f=a\,\cos \,\omega t$. (**a**) Chaos for Fig. 4(a). (**b**) Chaos for Fig. 5(a). (**c**) nonchaos for Fig. 7(a). (**d**) Critical behavior for Fig. 9(a).

The attractors we discussed so far are chaos, nonchaos and critical behavor according to their maximum Lyapnuov exponents being greater than zero, less than zero and oscillating around zero. These atrractors are very similar in many aspects including the phase diagram, time history, Poincare section and generation mechanism. The similarities can also be shown with the help of the transformation diagrams about the displacement *x* and periodical excitation in Fig. 10(a,b), that are the transformation diagrams of fixed-point chaos with respect to Figs 4 and 5. The transformation diagrams of the fixed-point strange nonchaotic attractor in Fig. 7 and critical behavior in Fig. 9 are shown in Fig. 10(c,d). These transformation diagrams indicate that every attractor possesses two different oscillations. One is periodic motion about the fixed equlibrium. The other is chaotic oscillation due to the time-varying equlibria, characterized by the “randomly” spiking oscillations with extreme sensitivity to initial conditions. So we will call these attractors as the peroidic-chaotic motion. When the chaotic oscillation is dominant, the maximum Lyapnuov exponent of the entire solution is greater than zero, and it is a chaos. If the periodic movement is much appparent, the maximum Lyapnuov exponent of whole solution is less than zero or oscillating periodically around zero. Hence, it is not a chaos.

## Conclusions

The periodic and chaotic motions have been found to coexist in the response of the Duffing system with time-varying linear terms over one period. Such a motion is a new phenomenon and can be an addition to the classic invariant sets to describe the complex dynamics of nonlinear systems. The periodic-chaotic motions such as chaos, nonchaos, and critical behavior are very similar in many aspects. It seems to be inadequate to use the maximum Lyapunov exponent alone to characterize these motions. Hence, the periodic-chaotic motions call for new methods to describe them.

## Data Availability

The simulation in the paper is based on the ode45 routine in Matlab, where the absolute and relative errors are 10^−5^ and 10^−10^ respectively.
